# Exercise, Sports, and Cardiac Rehabilitation Recommendations in Patients with Aortic Aneurysms and Post-Aortic Repair: A Review of the Literature

**DOI:** 10.3390/jcdd11120379

**Published:** 2024-11-27

**Authors:** Michael Stiefel, Hadassa Brito da Silva, Christian Marc Schmied, David Niederseer

**Affiliations:** 1 Department of Cardiology, University Heart Center, University Hospital Zurich, 8091 Zurich, Switzerland; michael.stiefel@usz.ch (M.S.); christian.schmied@hirslanden.ch (C.M.S.); 2 Hochgebirgsklinik Davos, Medicine Campus Davos, 7265 Davos, Switzerland; hadassa.britodasilva@hgk.ch; 3 Herzgefaesszentrum im Park, Hirslanden Klinik im Park, 8027 Zurich, Switzerland; 4 Christine Kühne-Center for Allergy Research and Education, 7265 Davos, Switzerland; 5 Center of Translational and Experimental Cardiology (CTEC), Department of Cardiology, University Heart Center, University Hospital Zurich, University of Zurich, 8091 Zurich, Switzerland

**Keywords:** aortic aneurysm, exercise, cardiac rehabilitation, thoracic aortic aneurysm (TAA), abdominal aortic aneurysm (AAA), aortic dissection

## Abstract

Introduction: Balancing the well-documented benefits of regular exercise, particularly its positive impact on cardiovascular risk factors like hypertension, with the potential risks for patients with aortic aneurysms presents a significant challenge. This narrative review aims to summarize the current evidence and guidelines to assist clinicians in making informed exercise and sports recommendations for patients with aortic aneurysms or post-aortic repair. Methods: Nine clinical trials on the effect of exercise on abdominal aortic aneurysms (AAAs) were identified, including one study on cardiopulmonary exercise testing (CPET) in AAA patients. As no clinical trials on exercise in thoracic aortic aneurysms (TAAs) were found, we extrapolated data from other studies on exercise in aortic diseases, including data from patients who have had an aortic dissection, as well as three studies on cardiac rehabilitation (CR) and one study on CPET after proximal aortic repair. Review articles and guidelines were also incorporated to ensure a comprehensive overview of the topic. Results: Currently, no clear correlation exists between intense sports activities and the development of aortic aneurysms or dissections. Conclusions: Light to moderate physical activity appears safe and beneficial for patients with aortic aneurysms and post-aortic repair. Given the lack of evidence linking athletic activity to aortic complications, caution is warranted in restricting such activities for athletes, underscoring the importance of shared decision-making. Regular follow-up and optimal management of cardiovascular risk factors are essential.

## 1. Introduction

Exercise is vital for health and preventing atherosclerosis, linked to aortic aneurysms and coronary artery disease, often called a ‘miracle drug’ for its extensive benefits [1]. However, increased blood pressure during vigorous activities makes physicians cautious about prescribing exercise to patients with (thoracic) aortic aneurysms due to the risks of exacerbating the condition or causing complications like rupture or dissection [2].

**Epidemiology of Aortic Aneurysms**: Aortic aneurysms, including thoracic (TAAs) and abdominal (AAAs), are significant health concerns. TAAs have an incidence of 5–10 per 100,000 person-years with a 0.2% prevalence and 1.6 per 100,000 annual rupture rate, often undetected during life [3,4]. In 2019, the global AAA prevalence was 0.9% among those aged 30–79, with 5–7.5% in men and 1.5–3% in women aged 65 years or older [5,6].

**Thoracic aortic aneurysms (TAAs)** primarily develop at the aortic root or ascending aorta, accounting for up to 60% of all cases. They have a diverse range of causes, including conventional cardiovascular risk factors such as hypertension, smoking, and aging, as well as atherosclerosis itself. Additional contributing factors include congenital anomalies (e.g., bicuspid aortic valve, aortic coarctation), genetic syndromes (e.g., Marfan, Loeys–Dietz, Turner), infections (e.g., syphilis), mechanical trauma, and inflammatory diseases (e.g., giant cell arteritis, Takayasu’s arteritis) [7]. Genetics is significant in about 20% of TAAs [4].

**Abdominal aortic aneurysms (AAAs)**, primarily driven by conventional cardiovascular risk factors, are influenced by smoking, male sex, family history of AAA, advanced age, hypertension, hypercholesterolemia, obesity, and cardiovascular conditions such as heart and cerebrovascular disease, and peripheral artery disease, as well as pulmonary and renal disease [5].

Despite being more prevalent in men, women with TAAs face higher risks of severe events like aortic dissection or rupture. TAAs are typically asymptomatic and often detected incidentally during unrelated imaging studies. The prevalence of both TAAs and AAAs has declined in recent years in developed countries, likely influenced by a reduction in smoking rates [4].

**Balancing the well-documented benefits of regular exercise**, such as its positive impact on cardiovascular risk factors, including hypertension, **against the potential risks for patients with aortic aneurysms** poses a significant challenge. This narrative review aims to summarize the current evidence and guidelines to assist clinicians in making informed decisions about exercise and sports recommendations for patients with aortic diseases.

## 2. Methods/Literature Search

In preparing this narrative review, a literature search was conducted using the PubMed database. The search focused on the potential benefits and risks of exercise in patients with aortic aneurysms, utilizing keywords such as ‘aortic aneurysm’, ‘thoracic aortic aneurysm’, ‘exercise’, and ‘rehabilitation’. Nine clinical trials on the effect of exercise on AAA were identified, including one study on cardiopulmonary exercise testing (CPET) in AAA patients. As no clinical trials on exercise in TAAs were found, we extended data from other studies on exercise in aortic diseases, including data from patients who have had an aortic dissection, as well as three studies on cardiac rehabilitation (CR) and one study on CPET after proximal aortic repair. Review articles and guidelines were also incorporated to ensure a comprehensive overview of the topic. An overview of the literature search details is provided in Table 1.

## 3. Results

### 3.1. Exercise and Cardiopulmonary Exercise Testing in Patients with AAA

Research on exercise for patients with small, pre-surgical AAA (up to 5.5 cm) shows safety and improved exercise capacity. Moderate exercise regimens, like cardiac rehab programs, enhance capacity without AAA-related events. Some studies, like AAA-STOP [9], found no growth rate differences between exercise and usual care groups, while Nakayama et al. [10] reported slower growth in the exercise group. Exercise may stabilize blood pressure and reduce inflammation, helping manage AAA expansion. Only four of the clinical trials on abdominal aortic aneurysms (AAAs) included a component of resistance training [9,11,12,13]. One trial involved low-intensity limb exercise training, while the others incorporated a short resistance segment of 10 min without specifying the intensity.

### 3.2. Specific Studies (See Table 2)

In 2018, Nakayama et al. [10] conducted a study of 49 rehab patients vs. 163 nonrehab patients prescribed a modified program to avoid excessive blood pressure during exercise. Their results showed lower AAA repair risk (a hazard ratio from 0.43 to 0.19) and slower expansion (2.1 ± 3.0 mm/year vs. 4.5 ± 4.0 mm/year). Elevated exercise blood pressure correlated with increased AAA expansion, suggesting benefits from controlled blood pressure.

In 2019, Nakayama et al. [2,11] conducted a study comparing 15 patients in a CR program to 25 controls. The rehabilitation group had a significantly slower AAA expansion rate (−1.3 ± 2.4 mm/year vs. 2.0 ± 3.6 mm/year, *p* < 0.01). The 150-day program included 1–3 weekly sessions of bicycle ergometer and low-intensity limb resistance training, with vital monitoring every 10 min to ensure blood pressure remained below 150/100 mmHg. Additionally, the program provided education on lifestyle, diet, and stress management.

In 2014, Myers et al. [9] carried out a study of 140 patients with AAAs up to 5.5 cm, in which 72 patients underwent exercise training, while 68 received usual care. Over 23.4 months, no adverse events or excessive AAA growth were observed in the exercise group, confirming the safety and efficacy of exercise training, which consisted of aerobic exercise aimed at burning 1000 to 2000 kcal per week, along with 10 min of resistance training per session.

In 2021, Niebauer et al. [14] conducted a substudy that showed that exercise training in patients with small AAAs significantly improved exercise capacity and beneficially affected vascular disease markers without accelerating aneurysm growth.

In 2018, Lima et al. [12] carried out a randomized controlled study, and 65 patients with small AAAs were assigned to either an exercise training group (n = 33) or a usual care group (n = 32). The exercise group participated in a supervised program consisting of aerobic and resistance training, with each session lasting approximately one hour, of which 10 min were dedicated to resistance exercises. The study found that exercise training improved ventilatory efficiency without accelerating aneurysm growth. There was no significant difference in the progression of the AAA between the exercise and usual care groups.

In 2012, Tew et al. [15] conducted a 12-week moderate-intensity endurance exercise program for 28 patients with small AAAs, and no adverse events or AAA size increases were reported. The study showed improvements in cardiopulmonary fitness and reductions in systolic blood pressure and high-sensitivity C-reactive protein.

In 2009, Kothmann et al. [16] showed that a six-week supervised exercise program (30 min of moderate intensity cycle ergometry, twice weekly) in patients with AAA significantly improved anaerobic threshold by 10% compared to usual care, though the mean benefit was below the minimum clinically important difference. Despite individual variability, the estimated number needed to treat for clinical benefit was five.

In 2017, Tew et al. [17] conducted a preoperative high-intensity interval training (HIIT) program for patients awaiting elective AAA repair, which was found to be feasible and acceptable despite the lower-than-intended exercise intensity.

In 2014, Barakat et al. [13] reported twenty patients slated for AAA repair participated in a six-week preoperative exercise program, showing significant improvements in peak VO_2_ (from 18.2 to 19.9 mL/kg/min) and anaerobic threshold (from 12.2 to 14.4 mL/kg/min), indicating enhanced cardiopulmonary fitness. Each one-hour session included six to eight exercises, such as heel raises, cycle ergometry, step-ups, arm weights, knee extensions, chair sit-ups, knee flexions, and graded treadmill exercises, with a 5 min warm-up and cool-down. Myers et al. [18], comparing AAA patients to age-matched referrals for CPET, found that AAA patients safely underwent maximal CPET with no serious events (Table 2).

**Table 2:** Clinical trials on exercise in patients with AAA.

Study Reference	AAA Diameter Population	Frequency (trainings/week)	Intensity	Time (min/session)	Duration (weeks)	Type or Comments
Nakayama et al., 2018 [10]	3.0–5.5 cm n = 212 (49 rehabilitation, 163 nonrehabilitation)	1–3	Anaerobic threshold, <~50% HRmax; BP < 150/100 mmHg	30	21	Moderate aerobic training; no strength training; sessions not started if resting BP > 130/90 mmHg; slower AAA growth rate vs. usual care, lower risk of AAA repair in the rehabilitation group
Nakayama et al., 2019 [2,11]	3.0–5.0 cm n = 40 (15 rehabilitation, 25 nonrehabilitation)	1–3	Below anaerobic threshold, BP < 150/100 mmHg	30	21	Bicycle ergometer training, low-intensity limb resistance training, vital parameters monitored every 10 min
Myers et al., 2014 [9]	2.5–5.0 cm n = 140	3	60–80% HRR, RPE 12–14	45	156	Hybrid home- and centre-based aerobic and resistance training; 1000–2000 kcal/wk goal; 10 min/session resistance training; no adverse events; 10% ↑ in VO_2_ at VT
Niebauer et al., 2021 [14] (subanalysis of Myers et al., 2014 [9])	<5.0 cm n = 96	3	Moderate intensity (60% HRR)	45 min endurance, 10 min resistance	52	Endurance and resistance training, improved exercise capacity, reduced lipid accumulation product, stable MMP-9 levels (vs. increase in usual care), similar AAA diameter growth in both groups
Lima et al., 2018 [12]	Small AAA n = 65 (33 exercise, 32 usual care)	3	Moderate to vigorous (60–80% HR)	60	12	Improvements for oxygen uptake efficiency and reduced cardiorespiratory demands during submaximal exercise
Tew et al. (2012) [15]	3.0–5.0 cm n = 28	3	RPE 12–14	35–45	12	Moderate endurance training; no adverse events; 94% adherence; 2.5 mL·kg^−1^·min−1 ↑ in VO_2_ at VT; reductions in hs-CRP and systolic BP
Kothmann et al. (2009) [16]	<5.5 cm n = 30 (20 exercise intervention, 10 usual care)	2	RPE 12–14 or moderate intensity	30	6	Moderate training; three dropouts, one due to cardiac arrest during exercise; no other adverse events; 10% ↑ in VO_2_ at VT
Tew et al., 2017 [17]	5.5–7 cm, preoperative n = 53 (27 HIIT, 26 usual care)	3	HIIT	10 min warm-up, 8 × 2 min intervals, 5 min cool down	4	Feasibility and acceptability confirmed, high exercise enjoyment, lower-than-intended exercise intensity
Barakat et al. (2014) [13]	Pre-surgery n = 20	3	Not provided	60	6	Aerobic and resistance training; 70% adherence; increases in peak VO_2_ (9%), VO_2_ at VT (18%), and exercise time (59%); no adverse events
Myers et al., 2011 [18]	3.0–5.0 cm 305 patients with AAA vs. 2155 veterans		CPET			Slightly higher hyper- and hypotensive responses in AAA patients; no serious events related to CPET in AAA patients

Abbreviations: AAA—abdominal aortic aneurysm; BP—blood pressure; CPET—cardiopulmonary exercise test; HRR—heart rate reserve; hs-CRP—high-sensitivity C-reactive protein; RPE—rate of perceived exertion; VO_2_—oxygen consumption; VT—ventilatory threshold [4].

### 3.3. Cardiopulmonary Exercise Testing and Cardiac Rehabilitation After Proximal Aortic Repair

Maximal Cardiopulmonary Exercise Testing (CPET) conducted three months post-proximal aortic repair on 128 patients revealed a significant 36% reduction in peak oxygen uptake (VO_2_ peak), indicating substantially reduced cardiorespiratory fitness (CRF). Importantly, all CPETs were performed without incident, underscoring the test’s safety [8].

For patients who have undergone surgical treatment for type-A aortic dissection, CR has shown promising results. Despite initial concerns about blood pressure increases, studies indicate that the hemodynamic responses to exercise in these patients are comparable to those in other cardiovascular patients. In a cohort of 19 patients post-type A aortic dissection, CR led to a 22% increase in VO_2_ peak, along with significant improvements in maximal workload and quality of life. The resistance training in the exercise-based cardiac rehabilitation program typically involved light to moderate weights. Exercises included movements such as arm curls, leg presses, and bodyweight exercises, focusing on major muscle groups to improve overall strength and functional capacity. The intensity was gradually increased as patients progressed, ensuring safety and effectiveness throughout the rehabilitation period [19]. A retrospective study of 73 patients demonstrated that early post-operative exercise-based CR is feasible and safe with close blood pressure monitoring. The resistance training intensity was moderate, tailored to each patient’s condition, and aimed to keep blood pressure below 160/100 mmHg. Adjustments were made based on perceived exertion and individual responses [20]. Another study involving 33 patients post-surgery reported substantial improvements in exercise capacity and work return rates, with few complications and no severe adverse events. However, two patients required further thoracic aorta surgery. Physical training programs included calisthenics, respiratory physiotherapy, walking, and cycling [21].

## 4. Discussion

There are no clinical trials for TAAs and only a few for AAAs, mainly due to concerns about exercise-induced blood pressure increases risking rupture. However, recent studies indicate no elevated rupture risk for AAA patients during exercise testing or training [9,10,13,15,16,22]. A total of seven trials incorporated resistance training that utilized light to moderate weights, including calisthenics, with intensity gradually increasing over time [9,11,12,13,19,20,21]. Patients with small, undetected AAAs (3.0–5.5 cm) in CR programs have shown improved function and risk factor modulation. Regular exercise may also provide hemodynamic benefits, potentially protecting the aortic vasculature against atherosclerosis progression.

### 4.1. Sports Activity, Aortic Dilation, and Dissections in Athletes

Height, sex, and age significantly influence thoracic aortic size [23]. Clinically significant aortic dilation is uncommon in younger athletes [23,24,25] but more prevalent in older endurance athletes [26]. Studies have shown that the average aortic dimensions in most athletes remain below critical thresholds [23,24], though taller athletes and older endurance athletes exhibit higher incidences of dilation. For example, 6–8% of volleyball players exceeded the established thresholds, while 31% of male master-level rowers and runners aged 50–75 years exhibited a dilated aortic root or ascending aorta [26,27].

Research indicates minimal variations in thoracic aorta size across different sports, suggesting that athletic activities generally do not significantly impact aortic root dimensions [28]. However, endurance athletes tend to have slightly larger aortic dimensions than power athletes, raising questions about the effects of high blood pressure during weightlifting [23].

Long-term studies on athletes with initial aortic sizes greater than 40 mm show only mild increases in aortic size with continued sports participation, with no significant cases of aortic dissection reported. However, three athletes experienced substantial aortic dilation (to 50, 50, and 48 mm) after 15 to 17 years in the absence of systemic disease, highlighting the importance of regular follow-up [23,24]. In patients with a bicuspid aortic valve, regular sports activity does not seem to affect aortic root size [29].

A notable exception is former American football players, who exhibit higher rates of aortic dilation (9% showing a dilatation of 45 mm or more), likely due to injuries from body collisions during their careers [30]. Conversely, a questionnaire study of aortic dissection survivors revealed that most individuals engaged primarily in low-intensity activities before their dissection, indicating that intense sports activities were not a common factor among those who experienced aortic dissections [31].

According to data from the questionnaire study, most patients (67%) were not exerting themselves physically or emotionally at the onset of their dissections [31]. Furthermore, a multicenter forensic study found that aortic dissection occurred during exercise in only 8% of cases, while 60% of dissections occurred during sleep or rest, implying that factors like sleep apnea or resting blood pressure may be more influential in causing dissections than transient increases in blood pressure during exercise [32]. Only 3 (0.9%) of the 357 reported athlete deaths between 1994 and 2014 in a large British cohort of consecutive cases of sudden athlete deaths were due to aortic dissection. The cohort had an average age of 29 years, and notably, 40% of all athletes died while at rest [33].

### 4.2. Arguments for Exercise in the Presence of Aortic Aneurysm

Exercise has significant benefits for patients with aortic aneurysms (AAAs), particularly regarding blood pressure, vascular function, and arterial stiffness. Both aerobic and repetitive weight programs effectively **reduce resting blood pressure**, achieving reductions comparable to antihypertensive medications (5–7 mmHg) [34,35]. The optimal regimen includes at least 30 min of moderate-intensity aerobic exercise daily, combined with dynamic resistance training 2–3 times per week, totaling at least 150 min weekly [36]. In addition, max. systolic BP during exercise showed no influence on growth rates of aortic diameters [37]. Higher-intensity exercise elicits a greater **anti-inflammatory response**, potentially protecting against aneurysm progression [38]. Moderate-intensity exercise improves flow-mediated dilation shortly after exercise in AAA patients, suggesting short-term vascular benefits. In contrast, higher-intensity exercise may pose short-term risks but stimulate longer-term vascular adaptations, such as **improved endothelial function** [39].

Exercise also **mitigates arterial stiffening** associated with aging. Studies show significantly lower arterial stiffness indices in endurance-trained athletes compared to sedentary peers despite similar blood pressures [40]. In AAA patients, exercise reduces arterial stiffness, with the greatest reduction following higher-intensity exercise, indicating its safety and efficacy [41].

Preoperative supervised exercise programs before elective AAA repair are **linked to improved long-term survival**, though they show no significant difference in long-term cardiorespiratory fitness or quality of life measures [42]. Reduced physical capacity prior to AAA repair predicts poorer survival [43], but **aerobic fitness improves** after supervised exercise [13].

Additionally, there is a **high prevalence of coronary artery disease in AAA patients**, with studies showing that 65% of AAA patients undergoing elective repair have significant coronary artery stenosis [44].

Moreover, exercise benefits extend to conditions like Marfan syndrome (MF). **In mouse models, moderate dynamic exercise moderates aortic root dilatation** without exacerbating aortic stiffness or causing histological damage [45]. Low-intensity exercise (55–65% VO_2_ max) offers the most protective effects on the aortic wall in these models [46].

Post-aortic dissection, regular aerobic activity is associated with significantly lower systolic blood pressure, emphasizing the need to address lifestyle and emotional changes in survivors to improve quality of life and reduce complications [31].

Overall, exercise improves exercise capacity and **beneficially affects vascular disease markers**, including lipid accumulation product (LAP, calculated using waist circumference and fasting triglyceride levels) and matrix metalloproteinase 9, without accelerating aneurysm growth [14].

### 4.3. General Management of Aortic Aneurysms

**Initial Diagnostic and Follow-up**: When an aortic aneurysm is detected, it is essential to evaluate the entire aorta and aortic valve at baseline and during follow-up, confirmed through CT or MRI [47]. The assessment should include family history, risk factors, Marfan syndrome scores, and often genetic testing [7,47,48]. Regular echocardiographic follow-ups every 6 to 12 months are crucial for monitoring the condition [7,23,49,50].

**Genetic testing**: Athletes with aortic dilatation should receive counseling and genetic testing if they have an aortic root diameter over 40 mm without a clear cause, features of connective tissue diseases, a family history of thoracic aortic aneurysm, dissection (under 60 years) or sudden cardiac death (under 45 years), or a bicuspid aortic valve with thoracic aortic aneurysm [51].

**Strict secondary prevention measures are recommended** for patients with aortic aneurysms due to their very high cardiovascular risk. Guidelines emphasize controlling risk factors such as smoking cessation and the optimal management of blood pressure, diabetes, and lipids. Patients should maintain LDL cholesterol levels below 1.4 mmol/L, similar to those with coronary heart disease, often requiring the use of statins [4,47,52].

**Medical management** of aortic aneurysms includes β-blockers and angiotensin II receptor blockers as primary treatments for thoracic aneurysms. Recent research highlights additional therapies like statins, metformin, and lifestyle modifications [53]. For AAAs, metformin, ACE inhibitors, angiotensin II receptor antagonists, and thiazides are linked to slower growth, emphasizing the importance of cardiovascular risk management and the potential of metformin in reducing aneurysm growth [54].

**Surgical intervention** is considered when the aortic root size exceeds specific thresholds: 45 mm for Marfan syndrome, 50 mm for a bicuspid aortic valve, and 55 mm for the general population [47].

### 4.4. Blood Pressure Response to Endurance and Resistance Training

During dynamic activities, the ascending aorta and aortic arch accommodate a 3- to 6-fold increase in blood flow and a significant rise in central pulse pressure (40–55%) [4]. In their 20s, systolic blood pressure (SBP) at rest averaged 117.9 mmHg, during treadmill exercise at anaerobic threshold averaged 138 mmHg, and at peak exertion averaged 182 mmHg, with values increasing with age [55].

Heavy resistance exercises cause extreme blood pressure (BP) increases due to mechanical compression of blood vessels, a strong pressor response, and the Valsalva maneuver. A small study measured BP responses in five bodybuilders during heavy weightlifting. Systolic and diastolic BPs rose rapidly during lifts, particularly during the double-leg press, which reached up to 480/350 mmHg in one subject. The Valsalva maneuver, indicated by mouth pressures of 30–50 Torr, contributed to these elevations [56].

### 4.5. Cardiac Rehabilitation for Patients with Aortic Aneurysms or Post-Aortic Repair

Patients with aortic diseases have complex care needs and benefit significantly from comprehensive CR programs. Evidence shows that thoracic aortic disease patients are often under-managed in terms of cardiovascular risk factors like blood pressure and lipid control [57]. Closer monitoring and support through ambulatory CR programs could improve outcomes for this high-risk group.

A study on a 12-week exercise program for patients with small AAAs revealed high compliance and positive feedback despite low recruitment, indicating the program’s manageability and benefits [58].

Thoracic aortic disease diagnoses often lead to diminished quality of life, exacerbated by recommendations to cease sports activities, causing stress and uncertainty [59]. Personalized guidance, including specific physical activity prescriptions and regular condition monitoring, is essential for managing health risks while maintaining quality of life. Comprehensive CR programs provide lifestyle and nutrition advice, optimized cardiovascular risk control (especially blood pressure monitoring), disease understanding, and psychological support. These programs are crucial for enhancing physical capacity, quality of life, and overall recovery for patients with aortic aneurysms and post-aortic repair.

## 5. Conclusions

The data suggest that continued athletic activity might not worsen aortic enlargement, but regular monitoring in patients with aortic aneurysms is essential. Older endurance athletes and those with frequent chest traumas may be at higher risk for developing an aortic aneurysm. To date, no clear correlation between intense sports activities and aortic aneurysms or aortic dissections has been shown, which raises caution in prohibiting such activities for athletes and emphasizes the importance of shared decision-making.

For patients with aortic diseases, certain training recommendations are important [49]. Moderate endurance and resistance training with light to moderate weights (e.g., calisthenics) seem to be beneficial and safe for most patients with aortic aneurysms and post-aortic repair. In resistance training, more repetitions with lower weights are preferred, and vagal maneuvers should be avoided. Contact sports should be avoided due to the increased risk of trauma.

The evidence consistently supports that moderate exercise regimens are safe and effective in improving physical function in patients with small, pre-surgical AAA. The most important aspect is the optimized control of cardiovascular risk factors through physical activity. These regimens can also prevent AAA expansion, likely due to reduced inflammatory activity, stabilized blood pressure during exercise, and improved endothelial function. CR remains an important strategy for preventing AAA expansion and rupture.

TAAs present more challenges, with less evidence on the effects of exercise. However, optimal blood pressure control and regular follow-ups are important. Personalized exercise recommendations should consider the underlying diagnosis, aortic diameter, family history, and individual fitness levels [49] (see central illustration). As various diseases may be the cause of the aortic aneurysm (i.e., Marfan, Ehlers–Danlos, inflammatory), we cannot speculate on the same benefits or risks for each.

For patients recovering from proximal aortic repair or type A acute aortic dissection surgery, CR programs are crucial. These programs enhance cardiorespiratory fitness and quality of life. It is recommended to maintain systolic blood pressure below 160 mmHg during both exercise tests and training sessions [60].

## 6. Future Directions

Future research should focus on the long-term outcomes of exercise interventions, particularly quality of life, cardiovascular events, and survival rates, in patients with TAAs and after type A aortic dissection. Additionally, leveraging wearable technology for continuous monitoring, mainly blood pressure, during exercise may enhance patient safety and management.

## Figures and Tables

**Table 1:** Literature search details.

Category	Details
**Databases used**	PubMed
**Inclusion/exclusion criteria**	
Study focus	Aortic aneurysms (including thoracic aortic aneurysms and abdominal aortic aneurysms), post-aortic repair (e.g., aortic dissection). Effects and safety of exercise in these patient groups.
Language	English
Study types	Clinical trials, randomized controlled trials, guidelines, systematic reviews
Selection process	Reading titles and abstracts; full texts were reviewed if helpful and available
**Search terms**	
Aortic aneurysm and exercise	572 results
Thoracic aortic aneurysm and exercise	110 results
Abdominal aortic aneurysm and exercise	289 results
Aortic aneurysm and exercise, clinical trial, and RCT	37 results (10 included, 6 duplicates, 20 not helpful)
Aortic aneurysm and rehabilitation	38 results
Aortic aneurysm and rehabilitation, clinical trial, or RCT	5 results (4 included)
Thoracic aortic aneurysm and exercise, clinical trial, and RCT	1 result (included [8])

## Data Availability

No new data were created or analyzed in this study.
